# A survey of a footwear service for people with Rheumatoid Arthritis (RA)

**DOI:** 10.1186/1757-1146-3-S1-P9

**Published:** 2010-12-20

**Authors:** Robert Field

**Affiliations:** 1 Bournemouth and Poole Community Health Services, Bournemouth, UK

## Background

Despite the DLF report ‘Footwear – a quality issue’ (1991), examples of poor usage of provided NHS footwear continue to be reported [1]. For footwear to be worn, design and fit to meet clinical needs plus the patient’s non clinical criteria have to be addressed [2]. Locally a specialist footwear service provides semi-bespoke and stock footwear to people with RA related foot pathology. Patients are fully included within decisions concerning provision. Service delivery is jointly by Shoefitter and Podiatrist.

## Method

A postal questionnaire was sent to 34 patients receiving footwear in 2009-10. Data from the 27 replies relating to the overall footwear service, quality and choice aspects to the provision, its usage and impact of provision is reported.

## Results

All were ‘very satisfied / satisfied’ with the clinic. Mean score of footwear characteristics (fitting; comfort; leather, colour, fastening and style choice) was 86.12/100. Mean footwear comfort was 8.65/10. 20 reported using footwear most/everyday. Since provision, 5 reported reduced podiatry need, 19 indicated changes in foot problems and 18 indicated improved activity participation.

## Conclusions

Results indicate high level of satisfactions with the footwear service and suggest a positive impact on individual’s participation in activities.
